# Pleiotropic Role of Tenascin-C in Central Nervous System Diseases: From Basic to Clinical Applications

**DOI:** 10.3389/fneur.2020.576230

**Published:** 2020-11-13

**Authors:** Chen Hanmin, Zhou Xiangyue, Cameron Lenahan, Wang Ling, Ou Yibo, He Yue

**Affiliations:** ^1^Department of Neurosurgery, Tongji Hospital, Tongji Medical College, Huazhong University of Science and Technology, Wuhan, China; ^2^Burrell College of Osteopathic Medicine, Las Cruces, NM, United States; ^3^Department of Operating Room, Tongji Hospital, Tongji Medical College, Huazhong University of Science and Technology, Wuhan, China

**Keywords:** tenascin-C, intracranial tumor, vascular diseases, neurodegenerative diseases, inflammatory, brain injury, therapeutic target

## Abstract

The extracellular matrix is composed of a variety of macromolecular substances secreted by cells, which form a complex network that supports and connects tissue structures, regulates the morphogenesis of tissues, and maintains the physiological activities of cells. Tenascin-C, a secreted extracellular matrix glycoprotein, is abundantly expressed after exposure to pathological stimuli. It plays an important regulatory role in brain tumors, vascular diseases, and neurodegenerative diseases by mediating inflammatory responses, inducing brain damage, and promoting cell proliferation, migration, and angiogenesis through multiple signaling pathways. Therefore, tenascin-C may become a potential therapeutic target for intracranial diseases. Here, we review and discuss the latest literature regarding tenascin-C, and we comprehensively explain the role and clinical significance of tenascin-C in intracranial diseases.

## Introduction

Tenascin-C (TN-C) was the first and most important member of the oligomeric glycoprotein family to be discovered. It is sparse in mature tissue, but its expression increases substantially under pathophysiological conditions, such as wound healing, angiogenesis, inflammation, and tumor formation. TN-C is central in regulating cellular proliferation, differentiation, migration, and apoptosis. Recently, substantial attention has been given to the role of TN-C in the occurrence and development of the central nervous system (CNS) diseases. Many articles have found that TN-C has a regulatory role in intracranial diseases, such as intracranial tumors, subarachnoid hemorrhage (SAH), and Alzheimer's disease (AD). Therefore, in light of its potential significance in the extracellular matrix (ECM), we will review published articles and comprehensively describe the crucial role and clinical significance of TN-C in intracranial diseases in this paper.

## Structure of Tenascin-C

TN-C is a glycoprotein with pleiotropic functions in the ECM and has a molecular weight of 180–300 kDa (1). Unlike the high expression found during embryogenesis, TN-C is sparsely expressed in mature tissue. However, it reappears in active sites of tissue remodeling, such as wound healing or cancer invasion (2).

**Figure 1:**
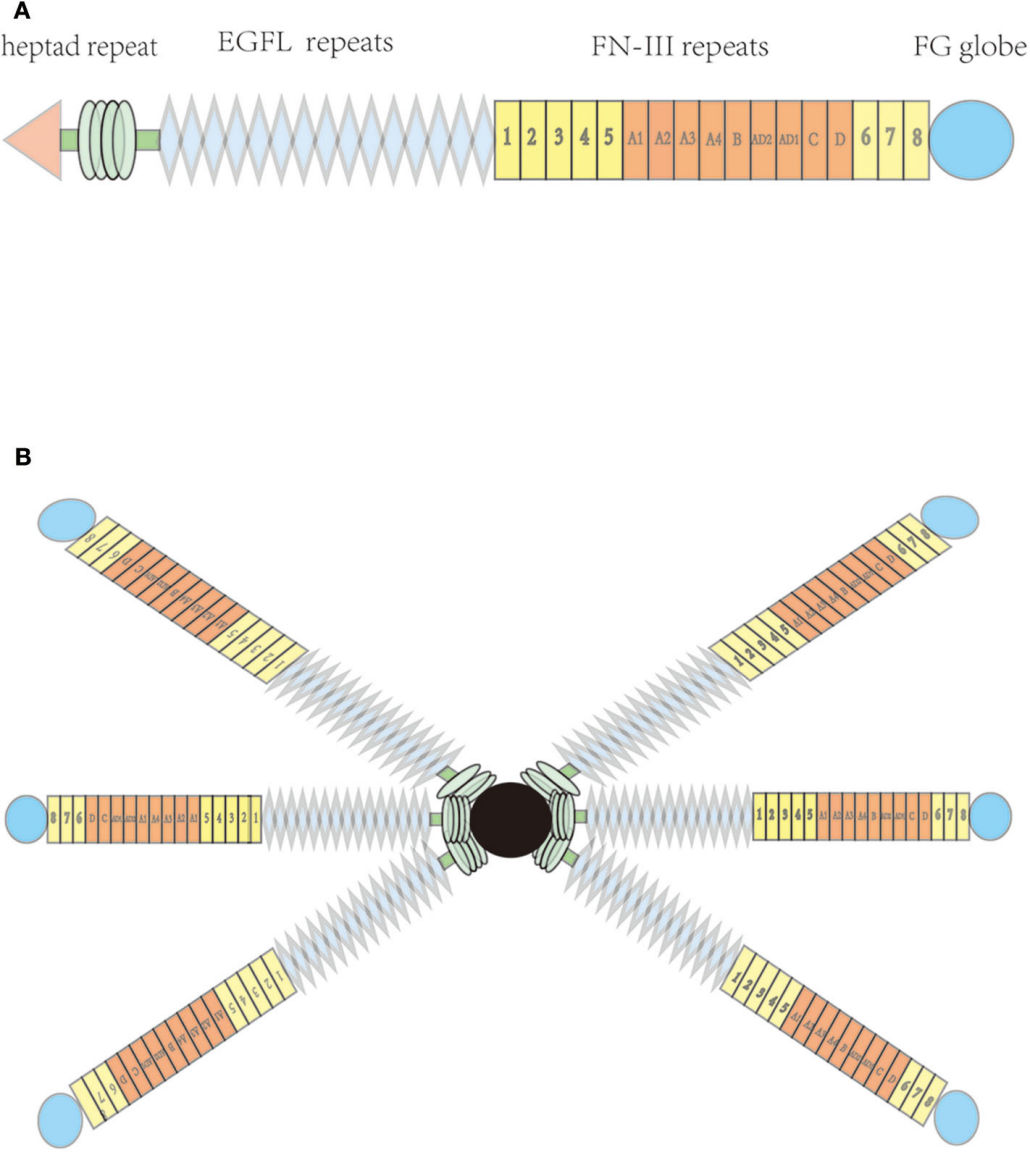
Molecular structure of tenascin-C; **(A)** Each subunit, including heptad repeat, epidermal growth factor-like (EGFL) repeats, fibronectin type III (FNIII) domains, and a C-terminal globular fibrinogen-homology domain **(B)** hexabrachion of the tenascin molecule.

TN-C is a six-armed structure composed of six identical subunits (3), known as hexabrachions. Each subunit includes a heptad repeat, a different amount of epidermal growth factor-like (EGFL) repeats, fibronectin type III (FNIII) domain repeats, and a fibrinogen-like globular domain (which is also present in fibrinogens) (Figure 1) (1, 3). Six cysteine residues, which are contained in each EGFL repeat sequence, may form intrachain disulfide bonds. Functionally, the EGFL regions in TN-C have an anti-adhesive effect on fibroblasts, neurons, and glial cells, which may promote neuronal migration (1) and provide localized signals for growth and differentiation (4).

All TN-C isoforms contain eight identical FNIII domain repeats. Six consecutive FNIII boxes located between the fifth and sixth FNIII domains can independently undergo alternative splicing, which may produce 64 different combinations, and may therefore encode TN-C proteins with different functions (5). Multiple ligands can bind to specific peptide motifs in the FNIII domain, such as various cell membrane receptors, mucopolysaccharides, and other ECM proteins (6). A fibrinogen-like globular domain can also reportedly bind to other ECM and cell surface proteins, such as collagenous fiber, heparin, integrin, and some proteoglycans (7).

## Function and Mechanism of Tenascin-C

**Figure 2:**
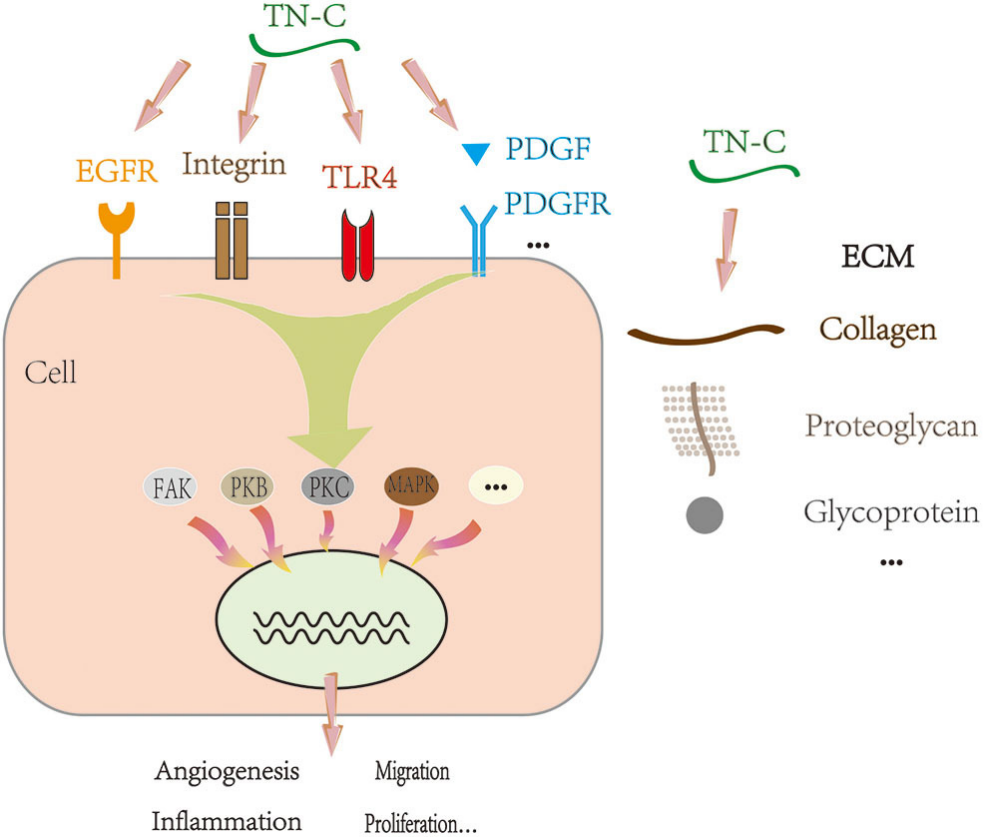
Proposed mechanism of TN-C under physiological and pathological conditions. EGFR, epidermal growth factor receptor; TLR4, Toll-like receptor 4; PDGF, platelet-derived growth factor; PDGFR, platelet-derived growth factor receptor; ECM, extracellular matrix; FAK, focal adhesion kinase; PKB, protein kinase B; PKC, protein kinase C; MAPK, mitogen-activated protein kinase.

TN-C can bind to cell membrane receptors, such as integrin, Toll-like receptor 4, and epidermal growth factor receptor. It may also bind to ECM molecules and cytokines, including brevican, neurocan, platelet-derived growth factor (PDGF) family members, transforming growth factor β superfamily members, insulin-like growth factor binding proteins, and heparin (8–11). By binding to molecules, TN-C can have a profound influence, exerting multiple effects, such as increasing cell migration and proliferation (12–14), downregulating focal adhesion integrity (15), promoting angiogenesis (16), altering ECM composition, and regulating gene expression (17). These processes depend on the activities of several classical intracellular signaling cascades (Figure 2), including the focal adhesion kinase, mitogen-activated protein kinase (MAPK), protein kinase B, and protein kinase C pathways (18, 19). For example, TN-C interferes with fibronectin signaling and blocks cellular adhesion by binding to syndecan-4 (20).

## Role of Tenascin-C in Intracranial Diseases

### Intracranial Tumors

**Figure 3:**
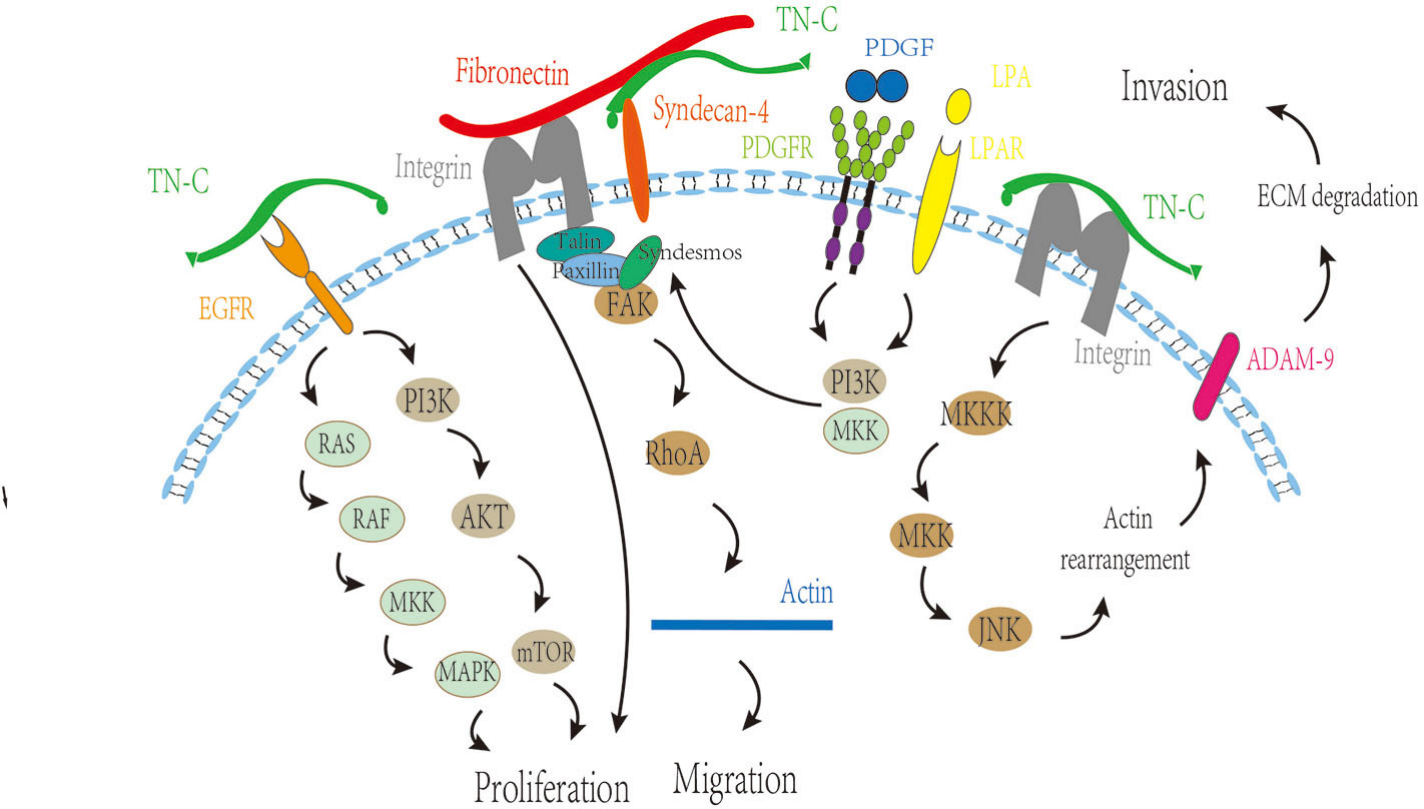
Regulatory function of TN-C on angiogenesis, proliferation, migration, and invasion of glioma cells. MKK, MAP kinase kinase; MAPK, mitogen-activated protein kinase; PI3K, phosphatidylinositol 3-kinase; mTOR, mammalian target of rapamycin; FAK, focal adhesion kinase; PDGF, platelet-derived growth factor; PDGFR, platelet-derived growth factor receptor; LPA, lysophosphatidic acid; LPAR, lysophosphatidic acid receptor; ADAM9, A disintegrin and A metalloproteinase-9; ECM, extracellular matrix.

The research of TN-C in intracranial tumors primarily focuses on glioma fields. There is a large amount of TN-C deposition in human gliomas, and TN-C is involved in angiogenesis, proliferation, migration, and invasion of glioma cells (Figure 3) (21).

#### Promotion of Angiogenesis

Recently, it has been reported that the expression of TN-C in the early stages of tumor progression is sharply upregulated, which is related to the promotion of angiogenesis (22). It was observed that TN-C was deposited in the blood vessels of high-grade tumors (23), and the alternatively spliced domains of the FNIII domains, TN-fnC and TN-fnA2, were also strongly expressed in tumor blood vessels (24–26). Not only was the expression of TN-C enhanced in high-grade tumor blood vessels, but also the TN-C antigenicity increased (23). Obviously, TN-C is highly related to tumor blood vessels (23, 26).

Vascular endothelial growth factor (VEGF) can promote endothelial cell proliferation and induce angiogenesis *in vivo*. Also, TN-C reportedly regulates VEGF expression and enhances its effects (27). In glioblastoma multiforme (GBM), perivascular TN-C strongly correlated with microvascular density and VEGF expression (28). After TN-C stimulation, GBM cells secrete pro-angiogenic factors, which are beneficial to endothelial cell survival and tube formation (22). Therefore, TN-C-induced tumor angiogenesis could be related to the binding and stimulatory effect of TN-C on endothelial cells (8).

#### Stimulation of Cell Proliferation

TN-C can stimulate cell proliferation (28). One known way to enhance tumor cell proliferation is to use TN-C to impair the adhesive function of fibronectin. TN-C accomplishes this by binding to the 13th FNIII domain of fibronectin, thus blocking the fibronectin-induced integrin signaling, leading to cell proliferation (20).

The special domain of TN-C is also related to cell proliferation. There are several domains, such as C, AD1, and AD2, which are located in the alternatively spliced region of FNIII domains, are highly overexpressed in the glioma, and also correlate with the proliferation rate of cancer cells (8). The N-terminal EGF type domains may be interrelated with the underlying mechanisms of tumor proliferation (10, 29, 30). The binding of the EGF domain to EGF receptors could activate these signaling pathways, such as phospholipase Cγ1, Ras/MAPK, and phosphatidylinositol 3-kinase (PI3K)/Akt pathways, thus increasing cellular proliferation (8). Interrupting the PI3K/Akt pathway by suppressing EGF receptor phosphorylation leads to decreased glioma cell proliferation (31, 32). Furthermore, the long fragment TNfnAll decreases cell proliferation, whereas the complete TN-C protein could increase the cell division index (26).

#### Promotion of Tumor Cell Invasion and Migration

It is generally accepted that TN-C leads to a hyperkinetic and invasive phenotype (33, 34). The alternatively spliced domains of TN-C are closely related to glioma cell migration (8). TN-fnA2 reportedly induces β1 integrin activation through syndecan-4 (35), and the processes of cellular spreading and focal adhesion formation are β1 integrin-dependent, which is fundamental for cell migration (36). In addition, TN-C regulates matrix contraction through modulation of focal adhesion kinase and RhoA activation (37), which changes the morphology and promotes migration.

The interaction between TN-C and growth factors could be an important mechanism to promote tumor cell invasion and migration. Human epidermal growth factor 2, a member of the EGF receptor family, can reportedly be phosphorylated, leading to receptor activation and glioma cell migration stimulation (38). We boldly propose that TN-C may be involved in this regulation process, acting by binding the EGF domain to EGF receptors. In addition, the interaction of TN-C and PDGF also plays a role in tumor cell migration. In a TN-C microenvironment, lysophosphatidic acid/PDGF signaling triggers glioma cell migration, depending on PI3K, Rho kinase, and MAPK/ERK 1/2, and revealed that a TN-C/lysophosphatidic acid/PDGF axis exists in malignant tumors (39).

TN-C mediates invasiveness in a metalloproteinase-dependent manner (40). Moreover, TN-C reportedly stimulates the invasiveness of brain tumor-initiating cells through interaction with a disintegrin and a metalloproteinase-9, and the mechanism involves the JNK signaling pathway (41). What is more, TN-C could modify glioma aggression through a positive feedback loop by directly binding to hypoxia-inducible factor 1-alpha (42). The metabolic enzyme, isocitrate dehydrogenase, is mutated in most low-grade gliomas and a small number of GBMs (43–46). Mutant isocitrate dehydrogenase 1 reduces ECM stiffness and mechanical signal transduction by downregulating HIF1α-dependent TN-C expression, thereby limiting gliomal invasion (42).

## Role of Tenascin-C in Vascular Diseases

**Figure 4:**
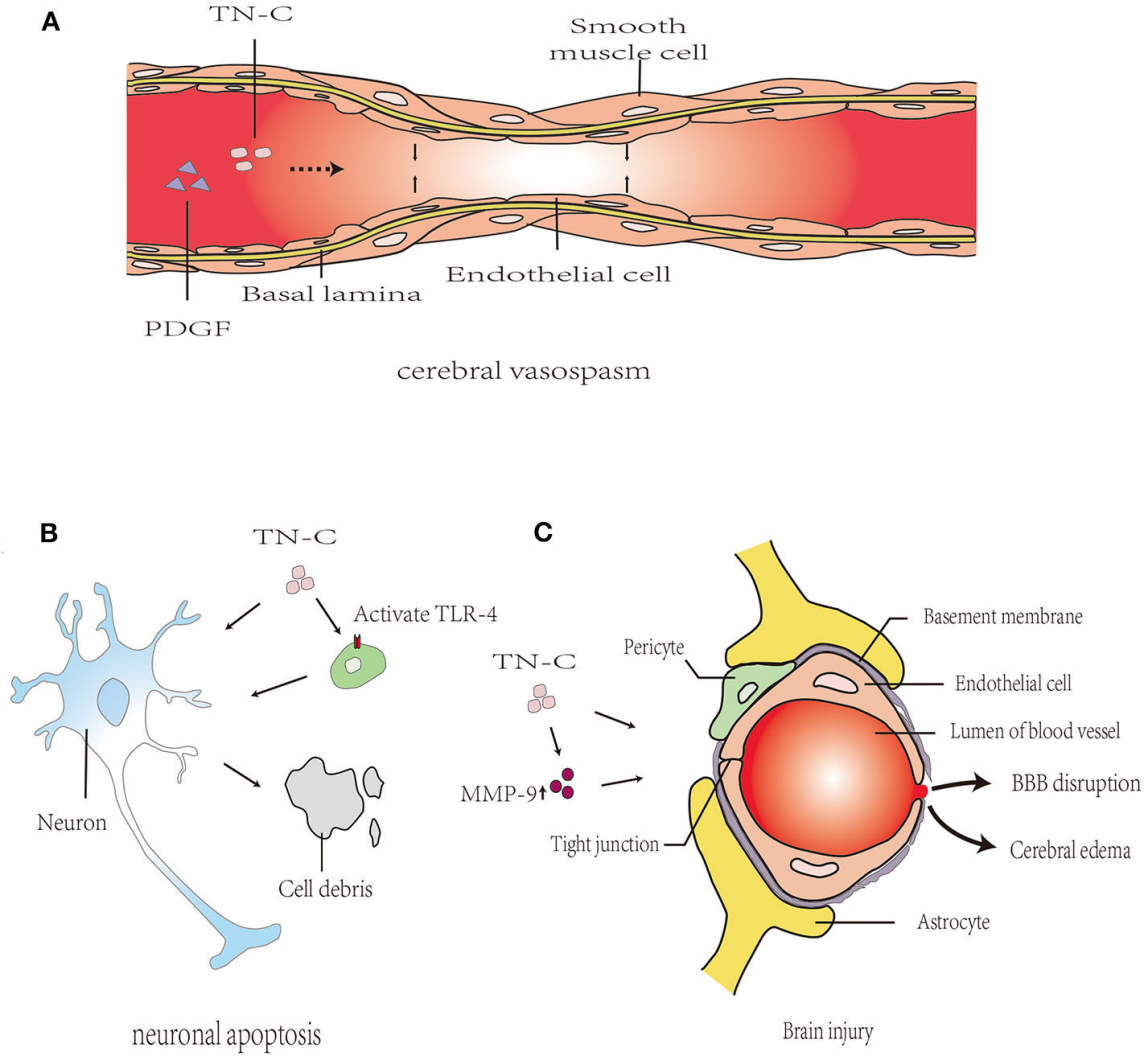
Proposed mechanism of TN-C in post-SAH brain injury. **(A)**: TN-C induced cerebral vasospasm after SAH via the upregulation of PDGFR-β. **(B)**: TN-C-induced neuronal apoptosis, partly mediated by TLR4/NF-κB/IL-1β pathways. **(C)**: TN-C induces brain edema and BBB disruption following SAH. PDGF, platelet-derived growth factor; PDGFR, platelet-derived growth factor receptor; BBB, blood–brain barrier; MMP, matrix metalloprotein; ZO, zonula occludens; JNK, c-Jun N-terminal kinase; ERK, extracellular regulated protein kinases; PI3k, phosphatidylinositol 3-kinase; MAPK, mitogen-activated protein kinase; TLR4, Toll-like receptor 4.

TN-C exists in the human circulatory system and reportedly participates in a range of cardiovascular diseases. In CNS vascular diseases, only one study found that the expression of TN-C in plasma was involved in large artery atherosclerotic stroke. Furthermore, TN-C could inhibit the release of pro-inflammatory factors from atherosclerotic arteries and could exert anti-inflammatory effects (47). In other studies, the role of TN-C in brain injury after SAH has become a hot topic in research. Blood–brain barrier (BBB) destruction, neuronal apoptosis, cerebral vasospasm, and delayed cerebral ischemia are the major contributing factors leading to brain injury (Figure 4).

### Blood–Brain Barrier Destruction and Brain Edema

The ECM components of cerebral microvessels in the basal lamina, such as collagen IV, fibronectin, and zonula occludens (ZO) proteins (e.g., ZO-1), were degraded by matrix metalloprotein (MMP)-9, causing BBB disruption after SAH (48, 49). TN-C induces brain edema and BBB destruction after SAH, which may be related to MMP-9 secretion and ZO-1 breakdown through the MAPK signaling pathway (50). In MMP-9 knockout mice, the degree of brain edema after SAH is significantly reduced, indicating that MMP-9 promoted the occurrence of brain edema, thereby participating in the progression of early brain injury (EBI) after SAH (51). In addition, the neuroprotective effects of TN-C knockout mice with SAH could be eliminated by injecting exogenous TN-C, thereby causing neurological dysfunction, BBB destruction, and brain edema.

### Neuronal Apoptosis

Neuronal apoptosis is associated with the pathogenesis of EBI after experimentally induced SAH (52). In the rat endovascular perforation SAH model, caspase-dependent neuronal apoptosis was found in the cerebral cortex 24 and 72 h after SAH and was associated with platelet-derived growth factor receptor (PDGFR) activation, MAPK activation, and TN-C induction (53). Neuronal apoptosis occurred at 24 h after injection of recombinant TN-C through the activation of ERK1/2 and p38 (53). In addition, the Toll-like receptor 4/NF-κB/IL-1β and IL-6 axis are also important pathways for TN-C to mediate caspase-dependent neuronal apoptosis (54). Therefore, the upregulation of TN-C expression after SAH is an important mechanism for neuronal apoptosis.

### Cerebral Vasospasm

Post-SAH cerebral vasospasms are reportedly caused by neuroinflammation and are characterized by the proliferation of smooth muscle cells (SMCs) and myofibroblasts, an altered phenotype of SMCs, intimal hyperplasia, cellular necrosis and remodeling, collagen deposition, and fibrosis (55–57). TN-C promotes SMC and myofibroblast migration and proliferation by reducing their interaction with the ECM, changing their cell phenotype, and contributing to protein synthesis (3, 58, 59). These reported functions of TN-C are consistent with the pathological changes of vasospastic arteries (60). After SAH, there is TN-C positive feedback in the cerebral vasospasm. SAH could upregulate TN-C expression in the cerebral cortex; meanwhile, the upregulated expression of TN-C caused post-SAH cerebral vasospasm by activating p38 and upregulating PDGFR-β (61). Therefore, we presume that the increased TN-C content in blood vessels after SAH may be related to the mechanism of vasospasm (60).

### Role of Tenascin-C in Alzheimer's Disease

**Figure 5:**
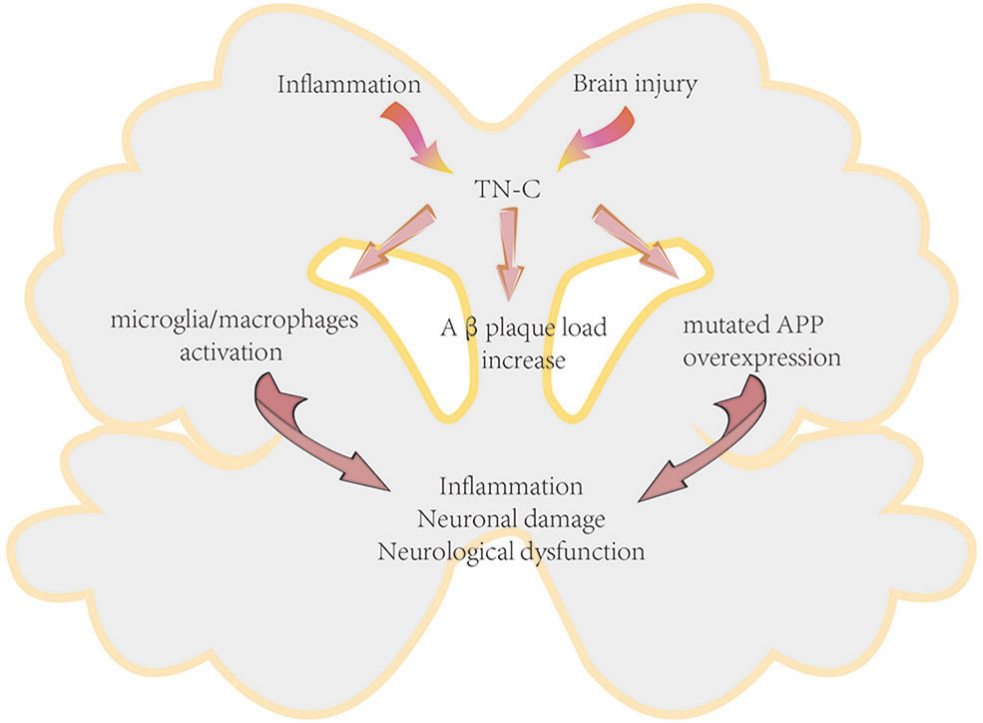
Proposed mechanism of TN-C in the pathogenesis of Alzheimer's disease. APP, amyloid precursor protein.

There are few studies regarding the effects of TN-C in AD. Recently, Xie et al. reported that TN-C is a molecule involved in enhancing chronic neuroinflammation in AD (62). Deficiency of TN-C could ameliorate the detrimental effects of mutated amyloid precursor protein overexpression, increase the number of microglia in the hippocampus and the adjacent cerebrum, shift neuroinflammation from a pro- to an anti-inflammatory pattern, reduce the cerebral amyloid β-protein (Aβ) plaque load, and protect neurons in AD mice, thus exerting beneficial effects on AD pathogenesis (Figure 5).

## Clinical Significance of Tenascin-C in Intracranial Diseases

TN-C is an important regulatory molecule involved in the occurrence and progression of intracranial diseases through multiple signaling pathways, indicating that it may become a therapeutic molecular target of new drugs, and an important biomarker and prognostic indicator.

### Tenascin-C as a Therapeutic Target for Drug Treatment

To date, some studies have confirmed the possibility of TN-C as a therapeutic target for drug treatment. For example, the A1 domain of TN-C is the specific binding site of the F16 human recombinant antibody, which was developed to target tumors (63). In addition, a new peptide, known as the Ft peptide, could synergistically target glioma-associated TN-C and neuropilin-1 in the neovasculature, circumventing the tumor's pathologic ECM barrier, and achieving deep penetration into the glioma parenchyma for anti-GBM treatment (64). Moreover, TN-C-induced glioma invasion could be attenuated by protein kinase C inhibitors, such as bisindolylmaleimide I, calphostin C, and rottlerin, through the downstream production of MMP-12 (65). Combining drugs that inhibit TN-C expression and chemotherapeutics may be a new treatment method for brain tumors because GBM neurosphere cells with TN-C knockdown are more sensitive to temozolomide (66). In addition, a randomized, double-blinded, placebo-controlled trial indicated that marimastat, a competitive and broad-spectrum MMP inhibitor, when used in combination with cytotoxic chemotherapy, has favorable effects in GBM patients (67). TN-C may be the underlying mediator in the trial due to the stimulatory effect on tumor invasiveness in an MMP-dependent manner. Furthermore, there is evidence that using radiolabelled anti-TN-C monoclonal antibodies during radioimmunotherapy may become a new therapy in treating brain tumors (68).

At present, an important treatment for SAH is to control vasospasm by maintaining blood volume and pressure, as well as using calcium antagonists, such as nimodipine. Efforts to develop more treatments that will improve the prognosis of SAH are necessary and important. With the in-depth study regarding the mechanisms of TN-C regulation, the upstream and downstream molecules of TN-C may be new therapeutic targets. Imatinib, a PDGFR inhibitor, was confirmed to alleviate neurological damage and vasospasm at 24–72 h after SAH in the rat model by attenuating the upregulation of PDGFR-β and TN-C (69). However, intraventricular administration of exogenous TN-C reactivated MAPKs and reversed the result of the anti-vasospastic, anti-apoptotic, and protein expression changes induced by imatinib (53, 69). Further TN-C molecular mechanism research and clinical trials can help us develop new therapeutic drugs for the management and targeted therapy of SAH. Cilostazol, an inhibitor of type 3 phosphodiesterase, inhibits intracellular cyclic adenosine monophosphate breakdown and has significant pharmacological effects, including vasodilation and antiplatelet function (70–72). A multicenter prospective endpoint trial reported that the application of cilostazol in the acute stage of SAH might be safe and effective. Cilostazol can prevent vasospasm and improve clinical outcomes for aneurysmal SAH patients receiving surgical treatment (73). Furthermore, cilostazol reportedly attenuates post-SAH cerebral vasospasm in rats (74), and its molecular mechanism may be related to the inhibition of TN-C expression through the protein kinase A system and MAPK pathway (75).

Although these drugs have significantly changed the development of intracranial tumors and vascular diseases *in vitro* and *in vivo*, they still require more rigorous clinical trials to support a formal clinical application.

### Tenascin-C as a New Biomarker and Prognostic Indicator

Recently, an increasing number of scholars believe that cancer stem cells (CSCs) are the root cause of malignant tumor recurrence and metastasis. Targeted therapy of CSCs brings new hope for the treatment of malignant tumors. Finding and identifying specific molecular surface markers of these CSCs are the keys to targeted therapy of CSCs. TN-C may be a novel marker for CSCs because TN-C+ cell populations have a strong ability to form spheres (76). Patients with malignant tumors usually have a poor prognosis, so early detection and early treatment are key to improving prognosis. Prognostic indicators can provide important clues for early tumor diagnosis, risk stratification, and patient management. Interestingly, the expression of TN-C is positively correlated with tumor progression, and the proliferation of tumor-supplying blood vessels is increased in the perivascular region (76, 77). Thus, TN-C may be used as a predictor of disease progression. In ependymomas, TN-C expression has been associated with anaplastic tumors (78). Using a candidate gene strategy, Puget et al. confirmed the feasibility of TN-C as a candidate gene for ependymoma progression in posterior fossa tumors (79). TN-C represents a marker that can help in the risk stratification of ependymomas (80). Tumors with a combined gain of 1q and TN-C expression reportedly have a poor prognosis. In contrast, tumors without a gain of 1q and TN-C expression have a favorable prognosis (79). In addition, tumors at different sites have a different expression of TN-C. The expression of TN-C is significantly higher in supratentorial and posterior fossa tumors than tumors found in other locations. The expression of TN-C is correlated with tumor grade, tumor location, and prognosis. Therefore, TN-C may help predict tumor progression and assist in the decision of treatment.

Although there are many methods for cerebrovascular examination to predict the occurrence of cerebral vasospasm, such as transcranial Doppler and angiography, they all have certain limitations, such as low accuracy or high cost. Therefore, it is necessary to develop more methods that accurately reflect the degree of vasospasm after SAH. It has been reported that TN-C levels in the cerebrospinal fluid (CSF) peaked immediately after SAH. Meanwhile, patients with symptomatic vasospasm had significantly higher TN-C levels than asymptomatic patients (81). Therefore, it is possible to detect the content of TN-C in CSF to predict whether vasospasm occurs after SAH. In addition, the serum TN-C concentration was positively correlated with the severity of trauma and poor clinical prognosis in traumatic brain injury patients but negatively correlated with the Glasgow Coma Scale (GCS) scores (82). Thus, TN-C is likely to become a new indicator to reflect the degree of brain damage after SAH.

Currently, AD is mainly diagnosed through clinical manifestations and pathological examinations but lacks specific indicators. Mi et al. found that TN-C deposits specifically surrounded the cored neuritic Aβ plaques, suggesting that TN-C may become a biomarker for AD and could be used as a diagnostic basis for AD neuropathological assessment (83). Furthermore, the TN-C levels of the CSF or blood may be used as diagnostic screening for AD to enable early intervention and better access to treatment (84).

## Perspective and Conclusion

TN-C expression is nearly absent in normal adult tissues, but when pathological changes occur, it increases dramatically. TN-C plays an important role in CNS diseases, working through multiple signaling pathways. In the tumor microenvironment, highly expressed TN-C can promote tumor cell proliferation, migration, invasion, and angiogenesis. In SAH, TN-C participates in the disruption of the BBB, induces neuronal apoptosis, promotes the occurrence of vasospasm, and increases cerebral edema. In AD, TN-C induces chronic inflammation and increases the load of Aβ plaques in the brain. Therefore, it may become an important therapeutic molecular target for the development of new drugs and may be used as a marker for disease classification, degree of injury, clinical prognosis, and to provide guidance for diagnosis and treatment strategies.

Technological advances in strategies targeting TN-C will allow us to conduct more translational studies on the prevention and treatment of TN-C-associated intracranial diseases. The drugs targeting TN-C proved to be efficacious in preclinical models, and clinical studies should be translationally applied to clinical practice. Because TN-C can bind to a variety of molecules, participate in multiple signaling pathways, and play a regulatory role in a variety of diseases, it is unknown whether targeted therapy of TN-C will cause additional pathological changes or unpredictable damage. Therefore, it is necessary to conduct thorough research on TN-C, especially to determine key points of the signaling pathway, which will provide a more theoretical basis for the development of novel methods in treating intracranial diseases.

## Author Contributions

CH wrote the article and made the figures. ZX, WL, and CL conducted manuscript revision in English. HY and OY participated in the correction and final review of this article. All authors read and approved to submit and publish the manuscript.

## Conflict of Interest

The authors declare that the research was conducted in the absence of any commercial or financial relationships that could be construed as a potential conflict of interest.
